# Exploring the Protective Effect of Food Drugs against Viral Diseases: Interaction of Functional Food Ingredients and SARS-CoV-2, Influenza Virus, and HSV

**DOI:** 10.3390/life13020402

**Published:** 2023-02-01

**Authors:** Andrea Ricci, Giovanni N. Roviello

**Affiliations:** 1 Studio Nutrizione e Benessere, Via Giuseppe Verdi 1, 84043 Agropoli, Italy; 2 Italian National Council for Research (IBB-CNR), Area Di Ricerca Site and Headquarters, Via Pietro Castellino 111, 80131 Naples, Italy

**Keywords:** SARS-CoV-2, coronavirus, gut microbiota, virus, herpes, HSV, infection, influenza, nutrients, immune system, functional foods, food drugs, phytochemicals

## Abstract

A complex network of processes inside the human immune system provides resistance against a wide range of pathologies. These defenses form an innate and adaptive immunity, in which certain immune components work together to counteract infections. In addition to inherited variables, the susceptibility to diseases may be influenced by factors such as lifestyle choices and aging, as well as environmental determinants. It has been shown that certain dietary chemical components regulate signal transduction and cell morphologies which, in turn, have consequences on pathophysiology. The consumption of some functional foods may increase immune cell activity, defending us against a number of diseases, including those caused by viruses. Here, we investigate a range of functional foods, often marketed as immune system boosters, in an attempt to find indications of their potential protective role against diseases caused by viruses, such as the influenza viruses (A and B), herpes simplex virus (HSV), and severe acute respiratory syndrome coronavirus 2 (SARS-CoV-2), in some cases mediated by gut microbiota. We also discuss the molecular mechanisms that govern the protective effects of some functional foods and their molecular constituents. The main message of this review is that discovering foods that are able to strengthen the immune system can be a winning weapon against viral diseases. In addition, understanding how the dietary components function can aid in the development of novel strategies for maintaining human bodily health and keeping our immune systems strong.

## 1. Introduction

The immune system consists of a broad range of mechanisms that provide defense against different pathogens. These defenses can be classified into two types: innate immunity and adaptive immunity, in which particular immune components work together to counteract infections. In addition to genetic factors that have an effect on human immune function, aging, lifestyle choices [1], and environmental determinants [2,3] can also affect our susceptibility to disease-causing agents. It has been established that certain foods contain chemical components that may regulate signal transduction, cell phenotypes, and pathophysiology [4]. Consuming specific probiotics and functional foods [5] may increase immune cell activity and defend us against different diseases, including viral infections, according to the scientific research [6,7,8].

Since ancient times, coloring, flavoring, and preservative agents have been created using aromatic herbs and spices. These latter elements, which have long constituted the cornerstone of traditional medicine in many countries, have been studied in particular by the pharmaceutical, chemical, and food industries [9]. Both in vitro and in vivo studies have demonstrated the anti-inflammatory [10], antioxidant [11], and several other properties of spices, such as hypolipidemic, digestive stimulant, antiviral, antimicrobial, and anticancerogenic [12] effects, suggesting the possibility to combat pathologies using these beneficial physiological outcomes. Viruses are known to be at the origin of a wide variety of illnesses, ranging from serious conditions such as cancer to minor respiratory ailments such as a cold [13]. Although the discovery of novel vaccines and targeted therapies has significantly lowered the mortality rate and severity of disease for numerous viral issues, there are still a significant number of viruses for which treatment or prevention options are insufficient, and recently emerging viruses can be very hazardous to human health. An example was given by the global coronavirus disease 2019 (COVID-19) pandemic, which seriously harmed the healthcare system and society globally, and may eventually endanger human lives, our prosperity, and our way of life. Several studies have indicated the beneficial antiviral properties of specific foods with respect to different ailments [14]. Some foods can strengthen the body’s defenses against viral infections by interfering with the viral life cycle or the host immunity [15]. The influenza viruses and the coronavirus SARS-CoV-2 [16], which causes severe acute respiratory syndrome and for which a number of prophylactics [17,18,19] and therapeutic strategies [20,21,22,23,24] are being exploited, are just a few examples of the viral pathogens that can be managed using foods with antiviral properties [14]. As rigorous testing is necessary for vaccines, quickly emerging viral respiratory tract infections (abbreviated as VRTIs) [25], particularly those caused by coronaviruses [26], often remain without an effective prophylaxis, significantly harming human health. In this context, functional foods can be supportive in preventing COVID-19 and VRTIs thanks to their antiviral and immunological activities, which are widely described in the scientific literature, highlighting the urgent need to identify new, immune-enhancing functional food components and their respective activities that could be controlled to combat viral infections and the consequent pathological states [27]. Given such a premise, in this review we aim to provide an overview of the practical benefits gainable from the ingredients of some functional foods, including fruits, spices, and aromatic herbs, and their dietary sources in the prevention and—where possible—therapeutic treatment of viral diseases, with a particular focus on COVID-19, influenza virus infection, and herpes. The primary mechanisms of action of the molecular components of some functional foods which regulate the action of the immune system or have a direct virucidal activity are also mentioned in our review. The final objective of this work is to show that discovering new methods for protecting health and preventing viral diseases can be facilitated through a better comprehension of the mechanisms of action of immune-enhancing functional foods.

## 2. Functional Food Effects on COVID-19, Influenza Virus, and HSV Infections

### 2.1. Functional Foods and SARS-CoV-2

COVID-19 is one of the most important pandemics that has infected the modern world. It is caused by the SARS-CoV-2 coronavirus, which mainly causes lung infections by binding to specific receptors present on the alveolar epithelial cells [28,29,30]. Due to the high homology with SARS-CoV [31], which caused SARS in 2002–2003 [31], structural, comparative studies with SARS-CoV [32] and the experimental and computational [33,34] screening of drugs which were somewhat effective with SARS and of natural compounds, especially from food sources [35,36], demonstrating anti-SARS-CoV activity, were among the first approaches adopted in the search for an effective anti-SARS-CoV-2 therapy.

#### 2.1.1. Plant-Derived Bioactive Compounds

**Figure 1:**
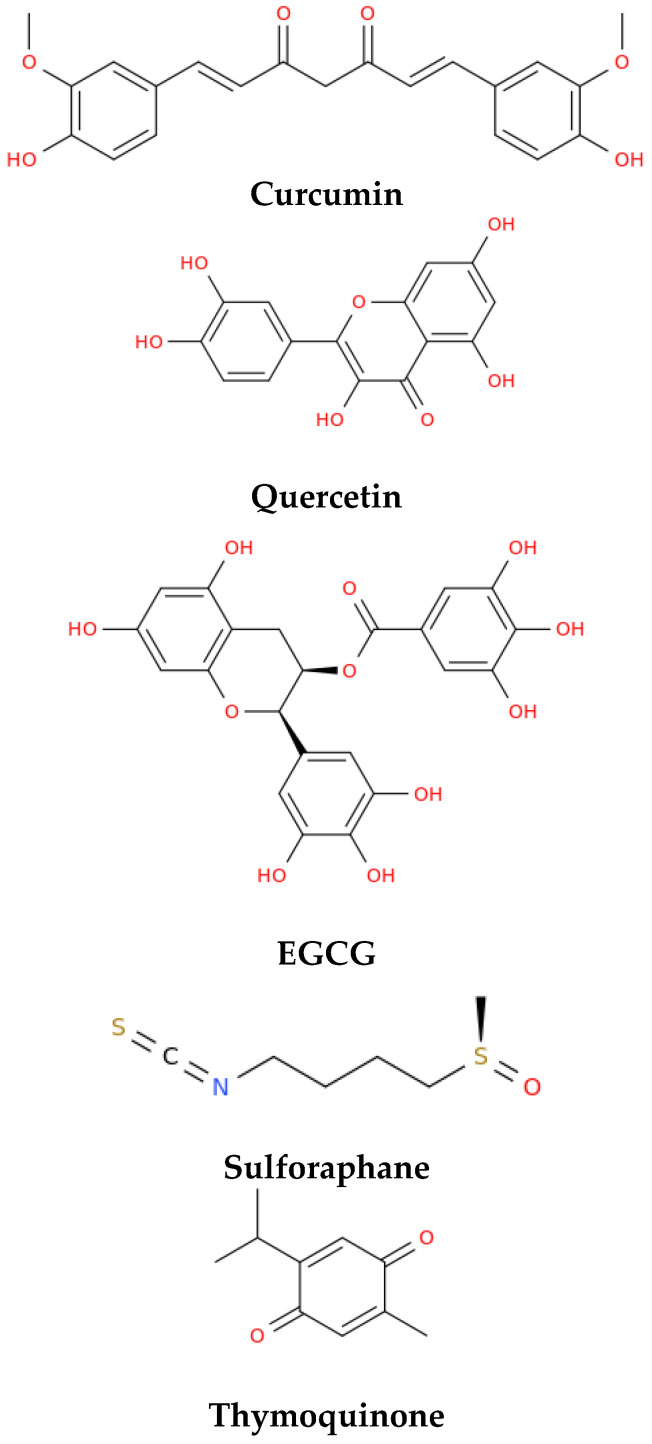
Structural representations of some of the main bioactive compounds contained in food supplements and traditional Chinese medicine products with activity against SARS-CoV which were investigated in the context of the disease caused by SARS-CoV-2.

The World Health Organization (WHO) organized an international conference in 2003 (https://apps.who.int/iris/handle/10665/43029, accessed on 12 January 2023) that was aimed at evaluating the results of different clinical trials using drugs in combination with various nutraceuticals and functional foods. Some of these remedies emerged from traditional Chinese medicine for their ability to prevent viral infections as well as modulate the body’s immunity. Below, we describe some of the bioactive compounds contained in food supplements and traditional Chinese medicine products that demonstrated activity against SARS-CoV and were subsequently investigated for possible analogous effects on the current SARS-CoV-2 virus in the context of the discovery of an effective COVID-19 therapy (Figure 1 and Table 1).

Quercetin [43] is present, among the other natural sources, in capers, onions, nopal cactus [44], and cloves, and exerts a significant antiviral effect against SARS-CoV and the H5N1 influenza virus [45]. Quercetin is regarded as a very effective drug for treating, mitigating, and preventing COVID-19 infection, especially if co-administered with other polyphenols, vitamins C, D, and E, and zinc [38]. Curcumin, prevalently found in turmeric, is active against different classes of viruses and, in the case of Coronaviruses, some studies have suggested its ability to bind the spike receptor-binding domain (spike RBD) and the angiotensin-converting enzyme 2 (ACE2) receptors [28,30], and the main protease of SARS-CoV-2, potentially inhibiting the virus [45]. Clinical trials conducted on hospitalized patients suggested that curcumin exerts its therapeutic effects by restoring the pro-inflammatory/anti-inflammatory nexus typically affected by COVID-19, and that its supplementation might represent an effective and safe choice for enhancing COVID-19 disease outcomes [37]. Epigallocatechin gallate (EGCG) is a phytochemical present in green tea. It demonstrates an activity against a large number of viruses and is able to bind to crucial receptors [45], preventing the virus RBD- human ACE2 interaction, which results in an attenuated SARS-CoV-2 infection [39]. Sulforaphane is a sulphur-containing compound, mainly found in broccoli, which has protective properties in the lungs and, through exerting an anti-inflammatory activity, is effective in coronaviral diseases by attenuating the effects of inflammation [46,47]. However, clinical trials are still needed to definitely prove the clinical efficacy of sulforaphane in COVID-19 patients [40]. Thymoquinone is a molecule present in black cumin (*Nigella sativa*) oil, which acts by blocking the replication of SARS-CoV [42]. This phytomolecule is considered a promising drug to treat several symptoms of COVID-19 and its related cardiovascular complications [41,42]. Sage polyphenols are natural compounds present in sage (*Salvia officinalis*), an aromatic plant commonly used in the Mediterranean diet. According to the scientific literature, some of them (such as rosmarinic acid and sageone) would be able to act as inhibitors of two key proteases for SARS-CoV-2 [33]. A significant activity of sage in reducing pulmonary fibrosis suggests that it can be used for therapy of COVID-19 and rehabilitation of post-COVID-19 patients [48]. Other compounds of dietary origin with a role in COVID-19 therapy include eugenol, clove phytocompounds [49], and resveratrol. This latter phenolic compound is found together with piceid (resveratrol-3-O-beta-glucoside) in grape juices. Both molecules are endowed with important biological activities, including their ability to interact with different DNA structures implied in cancer [50,51]. The antiviral potential of resveratrol was proven against a variety of viruses, including coronaviruses. In particular, this compound has been demonstrated to attenuate the main pathways involved in the pathogenesis of SARS-CoV-2, including the expression of ACE2 and the control of the renin-angiotensin system, the stimulation of the immune system, and the suppressed release of pro-inflammatory cytokines [52,53,54]. A rise in cytotoxic T lymphocytes and natural killer immune cells, as well as the promotion of SIRT1 and p53 signalling pathways, were also reported [52,53,54]. Resveratrol has also been proven to act as a powerful antioxidant by sequestering reactive oxygen species and as a stimulator of foetal haemoglobin. According to several literature reports, it may be also a potential therapeutic in the management of SARS-CoV-2 [52,53,54]. A recent, randomized, double-blind proof-of-concept study on mild COVID-19 patients, in addition to the encouraging results of other pre-clinical studies, indicated resveratrol as a potential drug for COVID-19 and possibly other viral respiratory diseases (such as human rhinovirus, influenza, and respiratory syncytial virus) [55].

#### 2.1.2. Gut Microbiota

In addition to the importance of integrating some functional foods into the diet, we must recognize the protective importance conferred to the human body (through the direct action on the immune system) by the microorganisms found in the human digestive tract (gut microbiota) [56,57]. An evident implication of the gut microbiota in COVID-19 was suggested by some hypotheses and subsequent observations that were published in the recent scientific literature [58,59]. SARS-CoV-2 RNA was also isolated from the faeces of infected patients [60,61]. Interestingly, cells of the intestinal epithelium, and particularly enterocytes of the small intestine, are also able to express ACE2 receptors [62]. On the other hand, the relationship of the gut microbiota to lung diseases has been well documented, and it was also demonstrated that infections caused by respiratory viruses cause perturbations in the gut microbiota itself [63]. Interestingly, nutrition, environmental determinants, and genetics all play a central role in determining the profile of the gut microbiota, which can, in turn, influence human immune responses. Intestinal microbial diversity decreases as we age and, interestingly, COVID-19 has been fatal mainly in older individuals. This once again suggests the impact of gut microbiota on COVID-19. However, it should be noted that, with age, numerous physiological activities of the body are weakened, and not just the diversity of the intestinal microflora. Nonetheless, improving the gut microbial profile through personalized nutrition [64] and appropriate supplements capable of improving the immune defences is one of the safest and most immediate prophylactic tactics. It can be combined with the currently available pharmacological therapies for COVID-19, aiming at minimizing the impact of the disease [65], especially in immunosuppressed patients and the eldest patients. Functional foods were reported to beneficially alter the gut microbiota; thus, the supplementation of nutraceuticals and trace elements could facilitate the improvement of the intestinal microbiota, particularly in COVID-19 patients with trace-element deficits, for which an abundant, diverse, and healthy gut microbiota not only reduces the severity of disease but was also suggested to increase the efficacy of COVID-19 vaccines [65].

### 2.2. Functional Foods and Influenza

The viral influenza infection [66,67] can have detrimental effects on global health when an outbreak takes place. In fact, influenza epidemics cause millions of hospital admissions every year as well as numerous fatalities. In the clinical setting, the infections are prevented using influenza vaccines [68,69] and cured with various drugs [70,71]. Despite the fact that there are several influenza virus vaccines and influenza drugs on the market and manufacturers modify the vaccine each year in response to the anticipated, mutated strain, the therapeutic and prophylactic strategies are still not very effective. The antigenic mismatch of vaccines and the emergence of drug-resistant viral strains, therefore, necessitate new strategies for treating influenza. Moreover, it is essential to prevent viral influenza infections, which can be aided by means of dietary supplements (Table 2) or any products that can fortify and shape our immune system [72,73]. A functional food consisting of a water-soluble combination of pomegranate, red grape, date, olive fruit, fig, and ginger extracts was developed in order to stimulate or modulate the production of particular cytokines and hemagglutinin inhibition (HAI) antibodies in mice after viral influenza vaccination [74]. By feeding the mice with this functional food mixture once intraperitoneally or multiple times orally for 1–7 days, it was possible to significantly enhance the production of interferon (IFN)–γ and interleukin (IL)–12 in their spleen, blood, and lungs [74]. When this mixture was administered orally for two weeks (one week apart) after two vaccine injections (secondary immunization), significantly higher titers of HAI antibodies were observed in mice than when it was administered orally for one week after a single vaccine injection (i.e., in the context of the primary immunization). Distinct and significant changes in IFN-γ production were associated with this rise in HAI antibodies. Notoriously, the immune defenses against a viral influenza infection are bolstered when functional foods are used [75]. Therefore, after the preclinical toxicology studies aimed at guaranteeing their safety, functional food mixtures can be assessed as potential products to be tested in clinical trials in order to determine, for example, their efficacy in minimizing the effects of influenza infection on humans [74]. In this frame, a study focused on natural foods with the main objective of developing novel influenza therapies did evaluate the juice of *Citrullus lanatus* (wild watermelon [76]) as potential anti-influenza agent [77]. This juice effectively inhibited the influenza virus replication in cellular models (Madin–Darby canine kidney cells [78]) and was found to inhibit viral adsorption and the late stages of viral replication in a time-of-addition assay, suggesting that the juice of the wild watermelon contains molecular components endowed with strong anti-influenza properties [77]. Viral adsorption analyses showed that the juice was capable of reducing the amount of viral RNA in cells at 37 °C but not at 4 °C, which was in line with the observation that the juice inhibits viral entry into host cells at 37 °C. These results suggested that the anti-influenza activity of the juice involved a mechanism other than the suppression of the viral attachment, which may explain the reduced viral internalization. After wild watermelon juice was administered into the nasal mucosa, mice previously infected with a mouse-adapted influenza virus also showed a significantly higher survival rate [77]. Thus, these and other previous findings [79] demonstrate the anti-influenza potential of wild watermelon both in vitro and in vivo, suggesting that its juice may be suitable for use as a functional food with anti-influenza applications [77,79]. It is known that peanut (*Arachis hypogaea*) skins, a low-value byproduct of the peanut [80] industry, contain high amounts of polyphenol molecules. One literature study tested the antiviral efficacy of the ethanol extracts of peanut skins against various influenza viruses in accurate, cell-based assays [81]. The higher the polyphenol content of the extracts, the higher the observed antiviral activity was, proving irrefutably that polyphenols are the key anti-influenza molecular components of the peanut skins. In more detail, the replication of the influenza A virus was effectively blocked by treatment with an extract made from roasted peanut skins, which showed a half maximal inhibitory concentration of 1.3 μg/mL. According to the same study, the extract prevented the influenza virus from replicating in its early stages, and both types (A and B) of influenza virus were susceptible to its activity [81]. Of particular note was the potent activity demonstrated by the extract against a 2009 H1N1 clinical isolate with a reduced sensitivity to the drug oseltamivir [82]. Moreover, the antiviral activity of oseltamivir and amantadine increased when combined with peanut skin extract, proving the potential of the peanut skin extracts in the development of novel therapeutic options for the treatment of influenza [81]. Haidari et al. examined the hypothesis that a pomegranate (*Punica granatum*) polyphenol extract was endowed with anti-influenza properties [83,84,85]. Making use of a real-time PCR, plaque assays—methods using drug-containing agarose overlays typically employed for demonstrating antiviral activities—and hemagglutination assays, the authors showed that the extract was effective in preventing the replication of influenza A virus [83] in MDCK cells, which are typically used as cell models in studies involving influenza viruses [86]. 

The pomegranate polyphenol extract demonstrated virucidal properties and blocked the agglutination of chicken red blood cells induced by the influenza virus [83]. The same work concluded that the pomegranate extract had a direct virucidal effect in addition to its ability to inhibit the replication of the viral RNA. However, it showed no influence on the movement of the virus ribonucleoprotein into or out of the nuclei of MDCK cells. Out of the four main polyphenols (caffeic acid, ellagic acid, luteolin, and punicalagin) detected in the pomegranate extract, punicalagin was found to possess distinctive anti-influenza properties, including a strong virucidal activity and a marked capability to not only prevent the viral RNA replication but also to reduce the ability of the virus to agglutinate chicken red blood cells [83]. Additionally, the synergistic interaction of the pomegranate polyphenol extract and oseltamivir increased the anti-influenza effect of the drug alone [83]. One of the diverse therapeutic traits of the biologically active cocoa (Theobroma cacao) constituents consists of their inhibitory effect on the influenza virus infection, demonstrated in a study performed using a cocoa extract realized by treating defatted cocoa powder with boiling water [87]. The extract caused a dose-dependent inhibition of the viral infection in MDCK cells that had been previously infected with avian influenza viruses (H5N1 and H5N9) and human influenza virus A (H1N1 and H3N2), and B (H3N2). Remarkably, the cocoa extract inhibited viral adsorption to the MDCK cells [87]. In studies conducted on animal models, the same extract markedly improved the survival of mice that were intravenously given a lethal dose of influenza virus. The same study included trials on patients in which the participants were split into two groups: one group consumed cocoa for three weeks before and after receiving the vaccine against the human influenza virus A (H1N1), while the control group did not. Although natural killer cell activity was increased in both, the cocoa intake group showed a more pronounced increase, suggesting that drinking cocoa strengthens the defenses against the influenza virus infection and the disease onset by enhancing natural and vaccination-induced immunity [87]. A Ginkgo biloba leaf extract was also able to affect the capacity of influenza viruses to infect MDCK cells [88]. Although plaque assays on agarose overlays containing Ginkgo biloba leaf extract revealed that the virus multiplication was unaffected after adsorption to host cells, the ability of the virus to infect cells was significantly reduced when the viral samples were pre-treated with the leaf extract, suggesting a direct action exerted by the extract on the virus particles [88]. However, no inhibitory effect was observed when the Ginkgo biloba leaf extract was given to the MDCK cells before they were infected with influenza viruses, excluding an influenza-preventive role of the ginkgo-derived agent. According to the hemagglutination inhibition assays in the same work, the leaf extract interferes with the interaction between the influenza virus and erythrocytes. In particular, influenza A (H1N1 and H3N2) and influenza B viruses were inhibited by the ginkgo biloba leaf extract [88]. Collectively, the above results suggested that ginkgo biloba leaf extracts contain one or more anti-influenza molecules that directly affect the vital properties of the influenza virus particles and prevent hemagglutinin’s ability to bind to host cells. Therefore, these findings not only supported the hypothesis of an antiviral utility of Ginkgo biloba [90,91,92] but also offered intriguing and important insights into the screening procedures that can be used in studies of anti-influenza virus activity [88]. Elderberry (*Sambucus nigra*) is one of the traditional natural treatments for respiratory problems [93,94] that was also recently investigated in in vitro and clinical studies [95,96]. Elderberry supplements have gained interest as a means of treating or preventing illness during the COVID-19 pandemic, but there was also concern that preparations from this plant might overstimulate the immune system and increase the risk of cytokine storms. Hence, both the benefits and drawbacks of elderberry for the treatment and prevention of viral respiratory infections were recently analyzed in a systematic review by Wieland et al., who also investigated the correlation between elderberry supplements and the unfavorable health effects brought on by an excessive production of pro-inflammatory cytokines [89]. Although there were no clinical studies connecting elderberry to effects related to inflammation, ex vivo cytokine production was found to occur in response to elderberry consumption [89]. Elderberry seemed to be able to lessen the severity of influenza, though the evidence was contradictory. In comparison to oseltamivir, an elderberry-containing product was associable with a lower risk of influenza complications and adverse events [89]. The study demonstrated that there is no connection between elderberry consumption and clinical outcomes related to inflammation, and that elderberry had some effect on inflammatory markers, even though this effect might fade with prolonged supplementation. A small study comparing elderberry and the nonsteroidal anti-inflammatory drug diclofenac provided some insight into the similar or slightly lower efficacy of elderberry in the long-term reduction of cytokines over time [89]. In summary, the scientific literature suggests that elderberry is potentially safe as a treatment for viral respiratory illnesses because there is no evidence that it overstimulates the immune system. Regardless, the evidence for the benefits and harms that the plant can cause constitute an ambiguous aspect that requires novel confirmations [89]. Widely present in vegetal species, isoquercitrin is known as one of the phytocompounds endowed with the highest antiviral properties against various viruses [97,98,99,100]. In particular, isoquercitrin [101] has a strong activity against the influenza A virus, as proven in a study evaluating the inhibition of hemagglutinin and neuraminidase activities in correlation with the anti-influenza properties of the molecule [102]. Green-fluorescent-protein-tagged influenza A (H1N1 and H3N2) viruses were employed to test the efficacy of isoquercitrin as an anti-influenza drug in studies based on fluorescent microscopy and fluorescence-activated cell sorting, showing that this compound significantly reduced the levels of green fluorescent protein expressed by influenza A virus infection [102]. This was consistent with the evidence that isoquercitrin lowered the cytopathic effects of the H1N1 and H3N2 influenza A viruses. Immunofluorescence analyses also revealed that isoquercitrin was able to inhibit the expression of the proteins of the influenza A virus. According to the results of time-of-addition assays, the flavonoid prevented viral attachment and entry and was also endowed with a strong virucidal effect when the virus was already present in cells [102]. The hemagglutination assay demonstrated that isoquercitrin had effects on the hemagglutinin of the influenza virus, while the neuraminidase activities of the influenza A viruses H1N1 and H3N2 were also markedly reduced by the flavonoid. Interestingly, this molecule inhibited the viral release, entry, and attachment, and was also endowed with virucidal properties, leading to the conclusion that isoquercitrin could be an effective antiviral agent to prevent influenza viral infections [102].

### 2.3. Functional Foods and Herpes 

**Figure 2:**
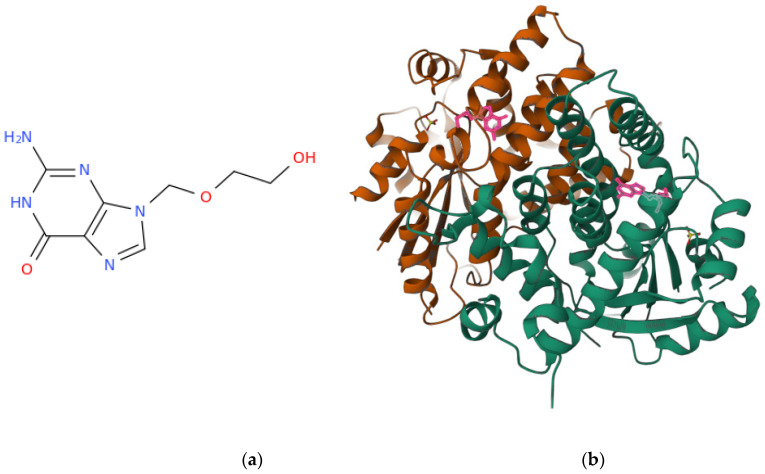
Acyclovir is an anti-HSV agent that is activated by the viral thymidine kinase to become inhibitor of the viral DNA polymerase: (**a**) structural representations of the HSV drug acyclovir and (**b**) dimeric complex of HSV-1 thymidine kinase in complex with acyclovir (pink). The complex structure is publicly available at the link https://www.rcsb.org/3d-view/2KI5/1 (accessed on 16 January 2023) and corresponds to the PDB ID: 2KI5 [115].

Herpes simplex virus (HSV) is a typical human virus that infects huge numbers of people globally, causing lesions that appear on the mucosa and skin of the mouth, nose, and genital areas, among the other body parts [103,104,105]. Sometimes serious or fatal HSV infections may also occur. In fact, HSV infection is significantly opportunistic, causing trouble especially in immunocompromised patients. As a result, in areas of the globe with a high prevalence of Acquired Immunodeficiency Syndrome (AIDS) [106,107] (caused by the Human Immunodeficiency Virus (HIV) [108,109,110]), such as sub-Saharan Africa, HSV infections have sharply increased, and herpes is often a severe illness. Different anti-HSV medications are on the market, but mutant viruses often emerge that are resistant to them and, in particular, to the first-choice drug for the type 1 of the virus (HSV-1), i.e., the nucleoside analogue [111,112,113] acyclovir (Figure 2a). From a molecular perspective, a clear role of thymidine kinase (Figure 2b) mutations in the insurgence of the acyclovir resistance was suggested by studies conducted on HSV-1 recombinant viruses [114]. Thus, it is critical to develop new, alternative treatments for HSV infections, especially those brought on by drug-resistant strains. In anti-HSV drug development, new drug side effects cannot be ignored. This explains the increasing scientific attention currently being paid to natural products with anti-herpes properties (Table 3). In fact, despite extensive investigations being made in the medical and pharmacological fields aimed at discovering effective synthetic drugs, plant and other natural sources such as functional foods remain a popular source of biologically active compounds to be used against HSV.

In this context, it has been demonstrated that the bioactive compound resveratrol, which can be isolated from grape juice and is endowed with different therapeutic properties [116], has a significant anti-HSV activity [117,118,119]. Regarding plant preparations that elicit anti-HSV effects [120], an aqueous total extract from climbing num-num (*Carissa edulis*) [121], a wild fruit and medical species used in Ethiopia and other parts of Africa, demonstrated remarkable anti-HSV properties in vitro and in vivo for both wild-type and drug resistant strains of HSV [122]. Endowed with a low cell cytotoxicity (CC_50_ = 480 μg/mL), the extract significantly decreased the formation of plaques in cells infected with wild-type or resistant HSV strains (HSV-1 and HSV-2) at a concentration of 50 μg/mL. The efficacy of the extract was also evaluated in vivo using mice that had been cutaneously infected with wild-type or resistant strains of HSV, revealing that at an oral dose of 250 mg/kg of the extract delayed the onset of the viral infections by over 50% [122]. Additionally, when compared to infected but untreated mice, the extract significantly increased the mean survival time of the treated, infected mice. In fact, when compared to the untreated, infected mice, which exhibited a mortality of 100%, the mortality rate for extract-treated mice was significantly reduced by more than 70%. Interestingly, no acute toxicity was found at the oral therapeutic dose (250 mg/kg). Overall, the above findings led to a hypothesis that the herbal extracts of climbing num-num are endowed with strong antiviral properties against herpes simplex viruses and could serve as an alternative therapy for herpes [122]. Among the functional foods with potential as antiviral remedies, propolis, a byproduct of honeybees, is endowed with relevant biological properties and medical applications [123], including a significant anti-HSV potential [124,125]. Several standardized propolis preparations with soy oil, glycol, glycerol, and ethanol were tested against HSV-1 and HSV-2 in a study aimed at determining the chemical composition and antiviral potential of each extract, using acyclovir as the control [126]. In particular, the glycolic propolis extract demonstrated an antiviral activity higher than acyclovir towards both HSV-1 and HSV-2, whereas preparations containing ethanol, glycolic acid, and soya oil had an antiviral activity higher than acyclovir only in the case of HSV-2 [126].

**Table 3:** Functional food ingredients used as anti-HSV treatments with the main corresponding natural sources and activities.

Functional Food Ingredient	Natural Source	Activity	Reference Number
Aqueous extract of climbing num-num	*Carissa edulis*	Effective in vitro and in vivo against both wild-type and drug-resistant strains of HSV-1 and HSV-2.	[122]
Propolis	*Apis mellifera* *	Glycolic propolis extract has anti-HSV-1 and HSV-2 activities higher than acyclovir; propolis leads to a delayed acyclovir cytopathic effect when it is co-administered.	[126,127]
Olive leaf extract	*Olea europaea*	Effective against HSV-1; when co-administered with acyclovir, it leads to a delayed cytopathic effect of the drug.	[127]

* Scientific name for the most-studied species of honeybees.

Propolis was also tested with an olive (*Olea europea*) leaf extract, individually or in combination with acyclovir, to determine their effects on HSV-1 [127]. Both functional food ingredients were found to be safe for human larynx cells and showed a significant antiviral activity when observed after incubations of 1 and 3 h at a concentration of 10 g/mL for propolis and 1.2 mg/mL for the leaf extract. There was a delayed cytopathic effect of acyclovir as a result of the combination of the propolis and the leaf extract with this drug, but no intrinsic cytopathic effects were observed. Overall, propolis and olive leaf extracts are effective against herpes on their own or in combination with acyclovir, and the co-administration may lessen the dosage and side effects of the reference drug [127]. This probably depends on the mechanisms by which the natural ingredients act in ways that are different from acyclovir, possibly through a direct virucidal activity, but also by the blockade of the virus internalization or the viral inhibition during the early stages of the replication of HSV-1. These extracts can be applied topically to treat acyclovir-resistant HSV infections, especially in individuals with compromised immune systems, and might also reduce the likelihood that oral lesions will spread and lead to infections [127]. 

Apart from the above-reported natural remedies for which evidence of antiviral activities have been demonstrated in the scientific literature, other plant-derived phytocompounds, such as garlic phytoncides and antiviral compounds of Mangifera extracts, deserve due attention in the fight against viruses [128,129,130].

## 3. Conclusions

Given the alarming rise in drug resistance and the limited efficacy of vaccines in the prophylaxis of new viral diseases, there is an urgent need to discover naturally occurring antiviral molecules and preparations that can be used as food supplements to bolster our bodily immunity. In this respect, there are a number of candidates for food drugs against COVID-19, even though conclusive proof of their efficacy has not yet been brought forward. Not less importantly, diet could also help reinforce immunity through the interaction that gut microbiota has with nutrition and different aspects of human health. Regarding influenza, there are a significant number of functional food ingredients, such as cocoa, pomegranate, and peanut preparations (to cite only a few), that are effective in combatting the effects of the influenza virus (both A and B strains) on human health. However, some food supplements, such as those based on elderberry, still require further investigations to definitely prove whether they can be included in anti-influenza functional foods. Among the anti-herpes agents available from dietary sources, it is worth mentioning propolis from honeybee as well as olive leaf extracts and, almost unknown in Western countries, the extracts of certain edible African plants such as *Carissa edulis*. Overall, the above-reviewed findings and considerations highlight the importance that a personalized diet rich in functional foods has in the fight against some of the most common viruses affecting humans in the current era. Additionally, the potential role of dietary supplements as preventative and therapeutic agents should be further researched.

## Figures and Tables

**Table 1:** Some of the chemical compounds ^1^ derived from functional foods studied for prevention or therapy of COVID-19.

Functional Food Active Compounds	Molecular Formula	Activity	Reference Number
Curcumin	**C_21_H_20_O_6_**	Alleviates the COVID-19 symptoms by restoring the anti-inflammatory and pro-inflammatory balance.	[37]
Quercetin	**C_15_H_10_O_7_**	With other polyphenols, vitamins C, D, E, and zinc, is effective in therapy and prevention of the disease.	[38]
EGCG	**C_22_H_18_O_11_**	Prevents the RBD-ACE2 interaction, which results in attenuated SARS-CoV-2 infection.	[39]
Sulforaphane	**C_6_H_11_NOS_2_**	May attenuate the effects of the inflammation.	[40]
Thymoquinone	**C_10_H_12_O_2_**	Alleviates COVID-19 symptoms and prevents the cardiovascular complications	[41,42]

^1^ These phytocompounds were previously evaluated against SARS-CoV.

**Table 2:** Some functional food ingredients used as influenza treatments with the main corresponding activities.

Functional Food Ingredient	Plant Source	Activity	Reference Number
Wild watermelon juice	*Citrullus lanatus*	Its juice effectively blocks influenza virus replication in cells and inhibits viral adsorption and replication.	[77]
Peanut skin extracts	*Arachis hypogaea*	Active against both types (A and B) of the influenza virus; peanut skin extracts prevent the influenza virus from replicating.	[81]
Pomegranate extract	*Punica granatum*	Pomegranate polyphenol extracts are effective in preventing influenza A virus replication and exert virucidal properties.	[83]
Cocoa extract	*Theobroma cacao*	Cocoa extracts dose-dependently inhibit the infection of different avian and human (A: H1N1, H3N2, B: H3N2) influenza viruses.	[87]
Ginkgo biloba leaf extract	*Ginkgo biloba*	Its leaf extracts directly affect the vital properties of the influenza virus (A and B) particles and prevent hemagglutinin from binding to host cells.	[88]
Elderberry supplements	*Sambucus nigra*	Elderberry supplements lower the risk of influenza complications and of adverse events by the long-term reduction of cytokines.	[89]

## Data Availability

Not applicable.
